# A Taxonomic Signature of Obesity in the Microbiome? Getting to the Guts of the Matter

**DOI:** 10.1371/journal.pone.0084689

**Published:** 2014-01-08

**Authors:** Mariel M. Finucane, Thomas J. Sharpton, Timothy J. Laurent, Katherine S. Pollard

**Affiliations:** 1 The J. David Gladstone Institutes, San Francisco, California, United States of America; 2 Institute for Human Genetics and Department of Epidemiology and Biostatistics, University of California San Francisco, San Francisco, California, United States of America; Charité, Campus Benjamin Franklin, Germany

## Abstract

Obesity is an important and intractable public health problem. In addition to the well-known risk factors of behavior, diet, and genetics, gut microbial communities were recently identified as another possible source of risk and a potential therapeutic target. However, human and animal-model studies have yielded conflicting results about the precise nature of associations between microbiome composition and obesity. In this paper, we use publicly available data from the Human Microbiome Project (HMP) and MetaHIT, both surveys of healthy adults that include obese individuals, plus two smaller studies that specifically examined lean versus obese adults. We find that inter-study variability in the taxonomic composition of stool microbiomes far exceeds differences between lean and obese individuals within studies. Our analyses further reveal a high degree of variability in stool microbiome composition and diversity across individuals. While we confirm the previously published small, but statistically significant, differences in phylum-level taxonomic composition between lean and obese individuals in several cohorts, we find no association between BMI and taxonomic composition of stool microbiomes in the larger HMP and MetaHIT datasets. We explore a range of different statistical techniques and show that this result is robust to the choice of methodology. Differences between studies are likely due to a combination of technical and clinical factors. We conclude that there is no simple taxonomic signature of obesity in the microbiota of the human gut.

## Introduction

Obesity is among the defining public health challenges of our time, with an estimated 3.4 million annual deaths attributable to high BMI [1]. Dietary and lifestyle interventions have only modest effects, and it is unclear whether these benefits persist over time [2]. Thus, there is substantial interest in alternative approaches to weight loss.

A tantalizing new theory has emerged in recent years, suggesting that the gut microbiome may offer a therapeutic target. Supporting a causal role of gut microbes in obesity, studies in mice showed that obesity can be induced in lean individuals via fecal transplants from obese individuals [3], [4]. While the mechanisms through which gut microbes influence BMI are unknown, multiple investigations of gut microbiome composition in both mice and humans have shown that obese individuals have a lower ratio of bacteria from the phylum *Bacteroidetes* to bacteria from the phylum *Firmicutes* than lean individuals [3], [5]–[7]. Obese individuals have also been shown to harbor less diverse bacterial communities [7], [8].

Both the scientific literature [9], [10] and the popular press [11] have heralded the association of obesity and the relative abundance of *Bacteroidetes* vs. *Firmicutes* as a robust finding. However, several recent reports question the strength of this association. Two large studies found no association between obesity and the *Bacteroidetes*: *Firmicutes* ratio [12], [13]. Furthermore, several publications actually report a *higher* ratio of *Bacteroidetes* to *Firmicutes* among obese individuals [14], in direct contradiction with the original findings.

The Human Microbiome Project (HMP) Consortium has collated the largest existing dataset describing the microbiota of healthy individuals, with sequences curated using stringent quality control. The cohort includes 16S rRNA sequencing of stool microbiomes from more than 200 adults living in Houston and Saint Louis [15], and it contains subjects with a comprehensive range of BMI values, including 24 obese (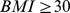) and 123 lean (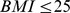) individuals. These data provide an opportunity to investigate the conflicting findings about taxonomic composition of the gut microbiome and obesity.

To this end, we conducted an extensive assessment of the relationship between BMI and the taxonomic composition of the gut microbiome in the HMP dataset and compared our results to trends in the MetaHIT data [16], which is another large survey of healthy obese and non-obese adults, as well as to two earlier studies that specifically sampled lean and obese individuals [6], [7]. Our analysis expands upon the work of the HMP Consortium, which explored a large set of candidate relationships between host phenotypic data (e.g., BMI, age, blood pressure) and microbial, enzymatic and pathway abundance and did not find a significant association between BMI and the relative abundance of *Bacteroidetes* or *Firmicutes* [13]. That analysis was designed to search automatedly over a large number of candidate relationships by a single pre-specified approach. To ensure that an association between BMI and gut community composition was not missed, we employed a range of graphical and statistical modeling techniques, quantified community composition in a variety of ways, and performed power calculations. This thorough interrogation of the data confirms that there is no association between BMI and stool microbiome taxonomic composition or diversity in the HMP cohort. When we examined the relationship between stool microbiome composition and BMI across different studies, we found that inter-study variability far exceeds differences in composition between lean and obese individuals within each study. Our results suggest that there is no simple relationship between BMI and gut microbiota and that significant technical and clinical differences exist between published studies.

## Results

### The *Bacteroidetes*:*Firmicutes* ratio is not associated with obesity or BMI

We began by attempting to reproduce the best-known result supporting the theory that obese individuals have a lower ratio of *Bacteroidetes* to *Firmicutes* in their guts. We found no difference between obese versus lean individuals in their relative abundance of *Bacteroidetes* or *Firmicutes* (p = 0.30 and 0.86, respectively, by 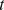-test).

Importantly, our failure to detect these differences was not due to insufficient statistical power. With our sample sizes and previously reported effect sizes, it is very unlikely that we would have found no association in the HMP data if an association did exist in the St. Louis and Houston populations. For example, using effect sizes from Turnbaugh et al. [7] for the V6 region of the 16S rRNA gene in European Americans, we would have had 96% power to detect a difference in the relative abundance of *Bacteroidetes* and 80% power for *Firmicutes*. These power calculations account for the fact that the proportion of obese individuals in the HMP cohort is lower than in [7].

**Figure 1:**
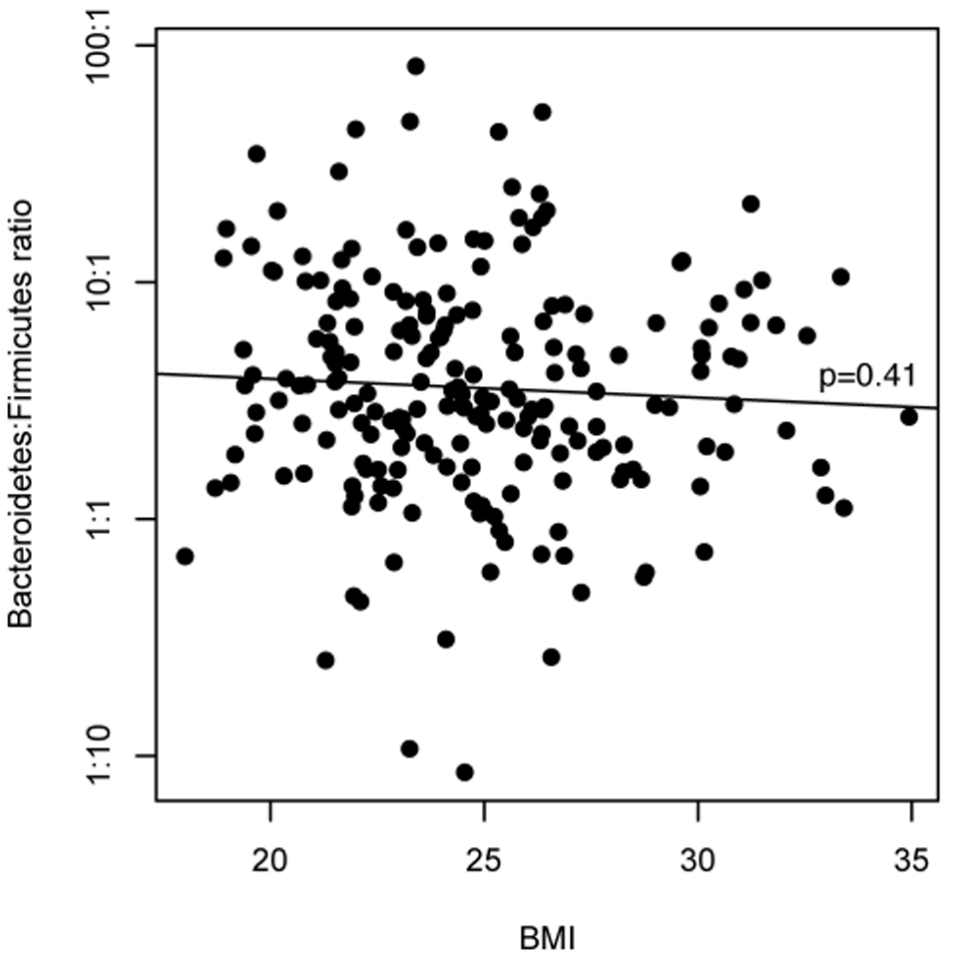
There is no association between BMI and the *Bacteroidetes*:*Firmicutes* ratio in HMP stool microbiomes.

Because the detrimental health effects of being overweight occur along a continuum of BMI values and not just above the obesity cutoff (BMI 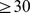), we next looked for a quantitative association between the continuous BMI variable and the ratio of *Bacteroidetes* to *Firmicutes*. There was no association (Figure 1; linear regression p = 0.41), and this ratio varied greatly between individuals regardless of BMI.

### Alternative quantifications of taxonomic composition are also not associated with BMI

**Figure 2:**
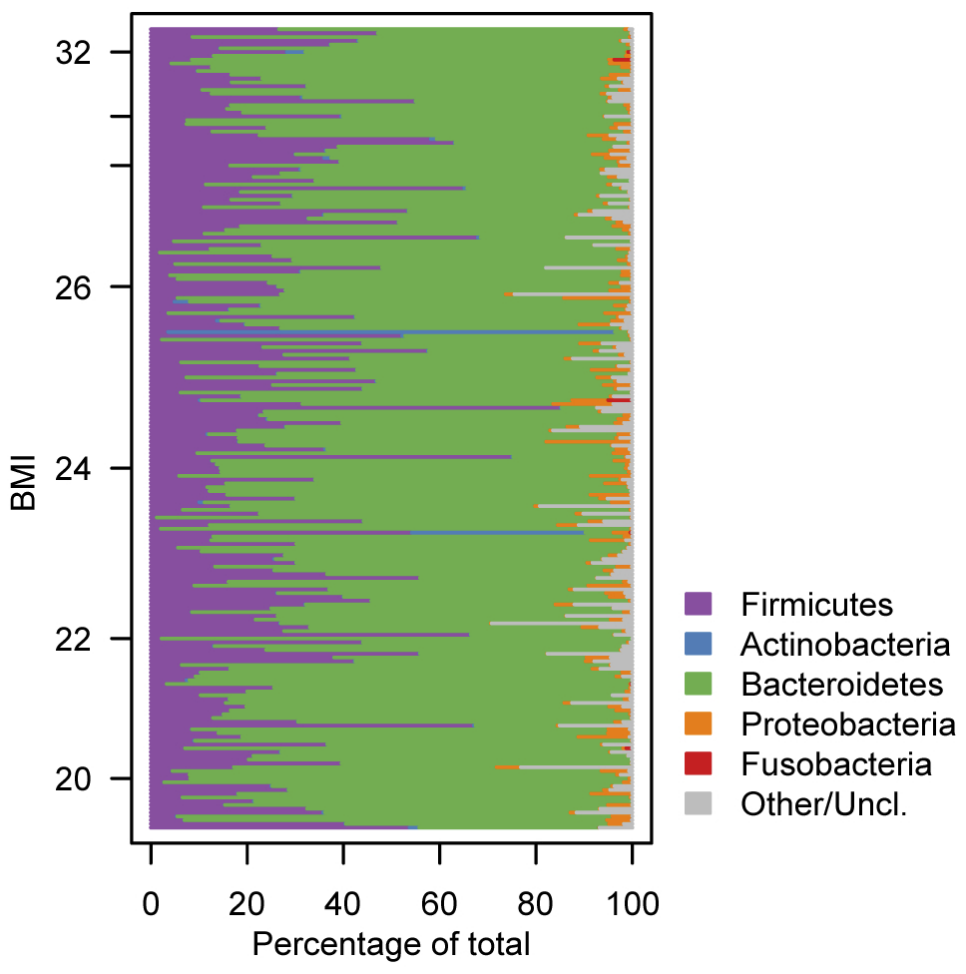
There is no relationship between BMI and the phylum-level composition of the microbiome. Each row shows the relative abundance of major gut bacterial phyla in an individual. Individuals are ordered according to their BMI.

Next, we investigated the possibility that – for the purposes of detecting an association with BMI – the ratio of *Bacteroidetes* to *Firmicutes* did not adequately summarize the taxonomic composition of the gut microbiome at the phylum level. Specifically, we quantified the relative abundance of the five major bacterial phyla in each sample and constructed a phylum-level compositional profile for each individual. We then visualized these compositional profiles as a function of BMI. No signal was apparent (Figure 2).

Then, to ensure that we had not missed a subtle pattern in this plot, we used a statistical model to isolate BMI effects from residual variance due to sampling and measurement error. Specifically, we modeled the isometric log ratio transform [17] of the relative abundance of each major phylum in each sample using a linear model, including a fixed effect of phylum plus a phylum-specific effect of BMI plus a random error term. We again found no significant association between BMI and taxonomic composition at the phylum level.

We then considered the possibility that a BMI association exists at a finer taxonomic resolution, despite the lack of association at the phylum level. For each individual, we generated a new taxonomic composition profile that quantified the relative abundance of each bacterial genus in the individuals stool microbiome. We applied principal components analysis to these genus-level profiles to reduce their dimensionality, as in Smith et al. [18]. We then tested for an association between BMI and any of the first six principal components (which explain 96% of the variance in genus-level profiles). We found no significant associations.

Additionally, to ensure that the principal components reduction did not obscure an association, we used logistic regression to model the probability of observing each major genus as a function of BMI. We again found no associations.

### Gut microbiome community diversity is not associated with BMI

Finally, we investigated the hypothesis that BMI is associated not with the relative abundance of particular taxa but rather the diversity of taxa present. This possibility was supported by Turnbaugh et al. [7] and by Le Chatelier et al. [8], who both concluded that a diverse gut microbiome can have a protective effect against obesity. Following the approach of Turnbaugh et al. [7], we used the 97% identity operational taxonomic unit (OTU) calls on the sequencing reads for each HMP sample to calculate rarefaction curves. We then used these curves to compare richness levels (i.e., total number of OTUs) between obese and lean individuals. In contrast to the results in [7] and [8], we found no relationship between richness and obesity, but rather observed a high degree of residual variability in OTU richness across individuals.

To ensure that these surprising results were not an artifact of diversity measure or calculation procedure, we validated our findings using the Shannon entropy measure as well as a variety of microbial ecology analysis software packages (mothur[19], QIIME [20], and MetaPhlAn[21]). This sensitivity analysis confirmed that our null diversity results were robust.

### Obesity effects are not consistent across studies

To ensure that we could reproduce the significant findings of previous analyses [6], [7], and to assess whether another large, recent study with a wider range of BMI values (MetaHIT [16]) could help clarify the contradictory results, we reanalyzed data from [6], [7], and from the Danish subjects in [16]. Although the primary analyses of the present manuscript were restricted to the V35 region of the 16S rRNA gene, we also included HMP data from the V13 region to assess the possibility that a 16S-region-specific bias could be obscuring a true underlying relationship.

**Figure 3:**
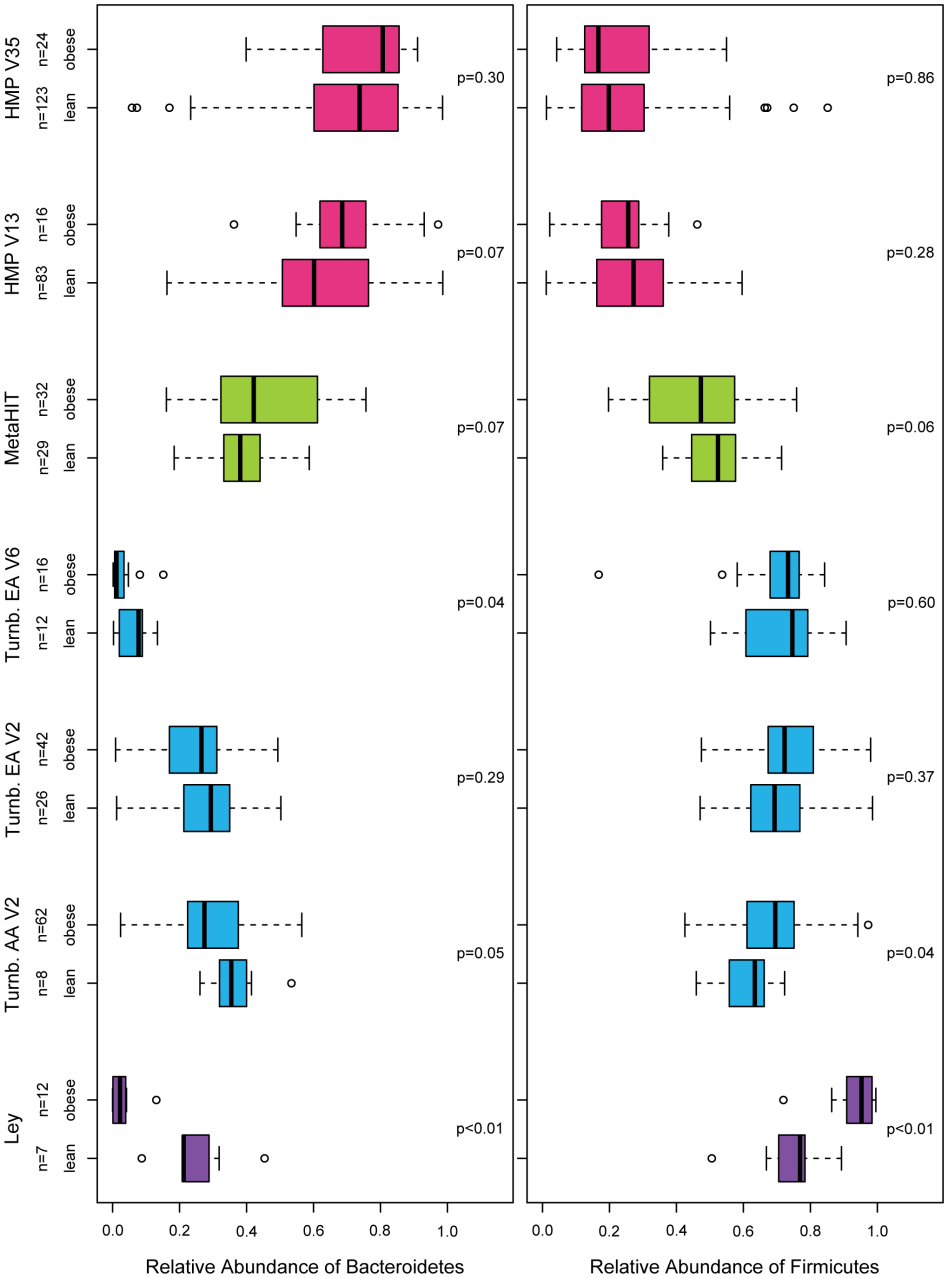
The between-study variability in the relative abundance of *Bacteroidetes* and *Firmicutes* is greater than the within-study differences between lean and obese individuals. The Ley data are from [6]. The “Turnb.” data are from Turnbaugh et al. [7], from African Americans (AA) and European Americans (EA), from variable regions (V) 2 and 6. The MetaHIT data are from the Danish subjects in [16] who do not have inflammatory bowel disease. The HMP data are from V13 and V35. We note that the primary results from this manuscript were generated using data from HMP V35. All p-values by 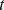-test.

In Figure 3, we show that variation in the relative abundance of *Firmicutes* and *Bacteroidetes* is much larger among studies than between lean and obese individuals within any study. Not only do the MetaHIT and HMP results fail to recapitulate the findings of Ley and Turnbaugh, but they actually go in the opposite direction. In the HMP data, this finding is consistent for the V13 and V35 regions of the 16 S locus, both of which were sequenced in the same individuals on the same platform (Roche 454). The substantial between-study variability could be due to an unmeasured factor such as diet [22] or due to technical factors such as DNA extraction technique, region of the 16 S locus targeted, or sequencing platform [23]. However, the consistent lack of BMI association for the V13 and V35 regions in the HMP data suggest that 16 S region is not a major confounder, at least in this cohort and for these two variable regions.

## Discussion

Fecal transplant studies in mice have shown conclusively that the microbiome has a causal effect on obesity [3], [4], and a number of high-profile papers have found that obese individuals have lower ratios of *Bacteroidetes* to *Firmicutes* [3], [5]–[7]. Yet we and others have found no relationship between BMI and the gut microbiome's phylum-level composition in large-scale analyses [12], [13]. These contradictory observations suggest that no simple taxonomic signature of obesity exists in the gut microbiome.

There are a number of possible reasons for these conflicting results. One possibility is that unmeasured confounders obscure a true underlying relationship. For example, diabetes status [24], diet [22], total caloric intake [25], or the duration of fasting periods [26] may be associated both with BMI and with microbiome composition. Thorough and standardized collection of host physiological data is needed to evaluate the contribution of these variables to the relationship between BMI and the composition of the intestinal microbiome. Another possibility is that technological issues may have differential effects across studies [14], [23]. Finally, it may be the case that the microbiome's effect on obesity is not mediated through its taxonomic composition but rather its function, since closely related taxa can have widely varying functions and distantly related taxa can have similar functions. This theory is supported by data from Turnbaugh et al. [7] (who found an enrichment of genes involved in carbohydrate and lipid metabolism in obese individuals), but not by results from the HMP Consortium [13] (who found no association between BMI and any pathway abundance). Additional functional metagenomic investigations are needed to determine whether a robust relationship exists between BMI and microbiome function. Once the nature of this relationship is better understood, further epidemiological work will be needed to estimate the proportion of human obesity attributable to microbial factors.

A limitation of our primary analyses is that they were restricted to the healthy subjects of the HMP cohort, none of whom had BMI 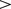 35. For example, we hypothesize that the HMP's health screen may have excluded low-diversity individuals, thus confounding our ability to discern an association between obesity and richness. We note, however, that the Danish MetaHIT cohort included 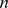 = 12 (17%) subjects with BMI 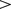 35, and it nonetheless revealed no association between obesity and the taxonomic composition of the gut microbiome.

## Methods

We downloaded high-quality, taxonomically annotated Roche V35 16 S rRNA gene sequences from the HMP Data Analysis and Coordination Center (www.hmpdacc.org). These are PCR-amplified V35 regions sequenced *en masse* on a Roche 454 instrument. These sequences were previously subject to extensive quality control analyses [15], including trimming, denoising, and chimera filtering. For each sequence, we extracted phylum-level taxonomic annotations and bootstrap statistics via RDP classifier 2.2 [27] using the default 032010 training set and taxonomy and the ‘allrank’ output format option, as per the HMP SOP. Sequences with annotations having a bootstrap statistic less than 80% were treated as “unclassified”. Sequences were then mapped to their corresponding HMP sample identifier and used to calculate phylum-level relative abundance for each sample. For subjects with multiple stool samples, since longitudinal BMI trajectory data were not available, we analyzed the sample with the largest number of reads. Out of a total of 217 available samples, we excluded five samples with 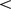
1000 reads, bringing our total sample size down to 212 samples. The same approach was used to obtain HMP V13 16 S rRNA gene sequences.

We tested for associations between arcsin square root genus relative abundance and all additional quantitative phenotypes from the HMP. We found no significant associations (FDR-adjusted 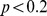) in any body site, consistent with the low number of microbe-phenotype associations found in [13] and supportive of the validity of our primary findings regarding BMI.

The MetaHIT data was composed of shotgun metagenomes, which we downloaded from The European Bioinformatics Institute (www.ebi.ac.uk). We restricted our analysis to the Danish samples that contained read-lengths of at least 75 base pairs (bp) (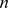 = 70), and we randomly analyzed 20 M reads from each sample. We quantified microbiome phylum-level diversity from these samples by using the STAP database [28] and the GreenGenes database [29] to identify metagenomic homologs of the 16 S locus and the RDP classifier to taxonomically annotate these sequences. We conducted a statistical simulation to identify the optimal bootstrap statistic thresholds for classifying 16 S RNA metagenomic reads into phyla using the RDP classifier. Briefly, we used Grinder [30] to simulate 10,000 75-bp 16 S reads from the STAP database and the GreenGenes database, plus 90,000 75-bp reads from the coding sequences (CDS) of 11 bacterial genomes randomly selected from the J. Craig Venter Institute?s Comprehensive Microbial Resource database [31]. All reads were subject to classification using the RDP classifier. We found that a bootstrap threshold of 70% captured 83% of the simulated 16 S sequences and correctly classified 99% of these reads while filtering out all but 0.001% of CDS reads.

Publicly available, high-quality 16 S amplicon sequences generated as part of the Ley [6] and Turnbaugh [7] studies were downloaded and taxonomically annotated. Specifically, we downloaded 18,348 full-length 16 S assembled from shotgun sequences generated in [6] from gordonlab.wustl.edu/microbial_ecology_ human_obesity/and 817,942 V6 hypervariable regions and 1,119,519 V2 variable regions of 16 S RNA pyrosequencing sequences generated in [7] from gordonlab.wustl.edu/NatureTwins_2008/. All sequences were classified into phyla using the RDP classifier as described above.
